# Health related quality of life (HRQOL) among low socioeconomic population in Malaysia

**DOI:** 10.1186/s12889-019-6853-7

**Published:** 2019-06-13

**Authors:** Sharifa Ezat Wan Puteh, Chamhuri Siwar, Mohd Azlan Shah Zaidi, Hazila Abdul Kadir

**Affiliations:** 1 Department of Community Health, Faculty of Medicine, Universiti Kebangsaan Malaysia, Kuala Lumpur, Malaysia; 2 Institute for Environment and Development (LESTARI), Universiti Kebangsaan Malaysia, Kuala Lumpur, Malaysia; 3 School of Economics, Universiti Kebangsaan Malaysia, Kuala Lumpur, Malaysia

**Keywords:** Bottom 40, Quality of life, Low socioeconomic status

## Abstract

**Background:**

The rapid growth of economy and increasing cost of living in Malaysia have given significant impact especially to the lowest household income population. The main objective of this study was to determine risk factors for low quality of life (QOL) and poor health status of this population.

**Methods:**

This was a cross sectional study design. A total of 347 respondents from low household income groups, including persons with disability and Orang Asli were recruited from E-kasih. A semi-guided self-administered questionnaire was used. QOL measured by EQ. 5D utility value and health status measured by visual analogue score (VAS). Descriptive statistic, bivariate Chi-square analysis and binary logistic regression were conducted to determine factors influencing low QOL and poor health status.

**Results:**

Majority of the respondents were Malay, female (61%), 63% were married, 60% were employed and 46% with total household income of less than 1 thousand Ringgit Malaysia. 70% of them were not having any chronic medical problems. Factors that associated with low QOL were male, single, low household income, and present chronic medical illness, while poor health status associated with female, lower education level and present chronic medical illness. Logistic regression analysis has showed that determinants of low QOL was present chronic illness [AOR 4.15 95%CI (2.42, 7.13)], while determinants for poor health status were; female [AOR 1.94 95%CI (1.09,3.44)], lower education [AOR 3.07 95%CI (1.28,7.34)] and present chronic illness [AOR 2.53 95%CI (1.39,4.61)].

**Conclusion:**

Low socioeconomic population defined as low total household income in this study. Low QOL of this population determined by present chronic illness, while poor health status determined by gender, education level and chronic medical illness.

## Background

Malaysia is a multiracial country with a total population of 31.7 million in 2016. The highest percentage distribution by ethnic group were Bumiputra (68.6%), followed by Chinese (23.4%), Indians (7.0%) and others (1.0%). Almost 70% or 22.0 million people from the total population belonged to the middle age group of 15 to 64 years old. There was a total of 7.6 million households with average of 4.1 persons per household [1]. Household income which is defined as total income received by members of households from four types of sources (income of paid employment, self-employed, income from property and investment and current transfer received) both in cash and in other forms of transfer which occur repeatedly within the reference period i.e. within a year, or more frequent. It showed an average monthly gross household income of Malaysian Ringgit (RM) 6141 in 2014 (equal to 1573.65 US Dollar for 31st December 2014) with an increase of 10.3% growth annually from the year 2007 (which was RM 3686) as reported by the economic planning unit [2].

Socioeconomic status (SES) can be viewed from the social and economic context, which is typically characterized by three dimensions namely education, employment and money [3]. There are few possible measures or indicators that can be considered to measure SES in order to decide the gradient level [4]. People from lower SES background tend to use public health services more than people from the high SES. It is worth considering whether this is a direct result from their generally lower income, or due to a broader social phenomenon. We can measure household income as a dimension for SES. Household income is a direct measure of capacity of people to purchase goods and services. Therefore, low level of household income could relatively be called low socioeconomic status.

The bottom 40% (B40) group which accounted for the 2.7 million household in Malaysia as reported in the Eleventh Malaysia Plan, has been explained by the growth rate in the welfare aggregate of bottom 40% [5]. This has been computed as the annualized average growth rate in per capita real consumption, or explained by the income of the bottom 40% of the income distribution in a country from household surveys roughly over a 5-year period. In this study, the B40 group has been categorised as the lowest household income group in which they are measured as a unit that earn a household income of RM 3855 (with currency rate of USD 892.26) and below in the year 2014.

World Health Organization (WHO) has recognized the importance of evaluating and improving quality of life (QOL) in all aspects of human life [6]. When QOL is related to health and disease, it is usually referred to as health-related quality of life (HRQOL). HRQOL has become an important measurement in healthcare and public health intervention. This widely used instrument consists of physical, mental, emotional, and social function measures. It is also related to the measurement of wellbeing and life satisfaction in relation to health status [7]. There are several instruments that can be used to measure HRQOL, and this study has been focusing on EuroQol five Dimension with three levels of problems (EQ. 5D-3 L) [8]. This questionnaire has been validated in Malay [9].

The rapid growth of economy in Malaysia with increasing cost of living is believed to have an impact especially among the lower SES population. This group of population has been the main target group by the government in helping them to increase their economy, income and wellbeing. The relation of SES and HRQOL has been widely discussed. The importance of socioeconomic conditions for health has been recognized since the early centuries [10]. Lower SES is usually correlated to the risk of having premature mortality due to non-communicable diseases [11]. Other studies found that housing conditions, health, and social support in poor urban setting had significant relationship with quality of life [12]. HRQOL measurement has been mostly used in clinical trial or intervention study, economic impact study in relation to cost utility analysis, as well as being a new measurement tool for health technology assessment study [13–15]. The importance of assessing HRQOL among the low SES population in giving inputs for decision maker for the benefit of the poorer group cannot be denied, especially for the purpose of improving their well-being and health as targeted in the National Plan.

The objectives of this study were to determine the HRQOL of low SES population in Malaysia and to explore the relationship between HRQOL with the demographic characteristics of this population.

## Methods

### Study design and data collection

This cross sectional study was a part of collaborative effort between Economic Planning Unit (EPU) Malaysia, United Nations Development Programmes (UNDP) and Universiti Kebangsaan Malaysia (UKM). Data collections conducted in August until September 2016, during an Open Space Technology (OST) forums reviewing B40’s views and perspectives on the rising cost of living issues which conducted in six main regions of Malaysia covering Northern, Eastern, Central, Southern, Sabah and Sarawak zones. In addition, two other specific OST sessions were conducted with B40 people with disabilities as well as with Orang Asli.

The participants were among Malaysian adults, aged 18 years old and above, with total household income of below MYR 3855 which selected among the E-kasih members from January 2011 to June 2016. The targeted number of total participant that has been decided by the UNDP were 720, comprising of 150 persons from central region and 100 persons from other 6 regions plus 35 persons with disabilities and 35 Orang Asli group. The actual total number of participant who joined the OST was 619. However, only 347 participants consented to complete the questionnaire. E-kasih was a poverty eradication portal, managed by the Prime Minister’s Department of Malaysia and a national poverty data bank which consists of information on the individual and household profile [16].

### Materials and study tools

A total of nine sections comprised in the questionnaire which has been used in this study consisting various aspect of B40 related information needed for the project. The data used in this paper were came from the first (respondent background) and the forth section (medical and health). The forth section which consist of the quality of life assessment using EQ. 5D-3 L self-administered questions.

EQ 5D was a standardised measurement tool for health status developed by the EuroQol Group to provide measure for health and quality of life in clinical and economic appraisal. HRQOL of the B40 participants has been assessed using EQ. 5D and EuroQol Visual Analogue Score (EQ-VAS) for the health status. EQ-VAS records the respondent’s health on the same day on a vertical visual analogue scale from 0 to 10, in which 10 is labelled as ‘best imaginable health’ and 0 score is the ‘worst imaginable health state’ as rated by the respondents. EQ. 5D-3 L has been validated among Malaysian adult population with an acceptable concurrent validity of EQ. 5D with SF-12 questionnaire (r = MCS-12: 0.2 and PCS-12: 0.4) (9). The reliability of test retest of the EQ. 5D showed a range of Intraclass Correlation (ICC) between 0.01 to 0.92 for the 5 dimensions in EQ. 5D, and validity of Spearman Rank correlation coefficient of Malay, English, Mandarin, and Tamil ranged from 0.61 to 0.86 [17].

The EQ. 5D descriptive system comprises of five dimensions: mobility, self-care, usual activity, pain discomfort and anxiety/ depression in which each of the item explained the problem or issue on the same day. Each dimension has three levels of score i.e. score 1 (no problem), score 2 (some or moderate problems), and score 3 (extreme or severe problem). Each respondent will give five numbers of scoring representing each dimension from the EQ. 5D Questionnaire. An example of 11,111 score, which means that the respondent did not have any problem with all the dimensions or equal to full health status, while 33,333 denotes severe problem for all dimensions which could be equal to death [18]. The EQ. 5D-3 L scores were then translated into HRQOL utility value, ranging from 0.0 to 1.0 (the nearest the value to 1.0, signifies better HRQOL), adapted from the Malaysian setting valuing method for EQ. 5D score [19, 20]. Variability in interpreting HRQOL were influenced by the populations studied [21, 22].

#### Independent variables

There were eight domains for independent variables; age, place of living, ethnicity, marital status, educational level, employment status, total household income and medical illness status. Each variable classified into two groups of categorical data. The mean value for the continuous data (age and household income) used as cut off point between two groups.

#### Outcome

There were two categorical outcomes in this study. The HRQOL outcome measured by the EQ. 5D-3 L utility value score and the Health Status measured using EQ-VAS. The mean value score of the two outcomes were measured and categorized into two groups by mean value as cut off point; high and low (HRQOL and Health status).

#### Statistical analyses

All data were analysed using statistical software SPSS package version 23. A two-tailed *p*-value of < 0.05 was considered as statistically significant. Statistical test by descriptive and binary logistic regression were done to analyse the outcome measured.

## Results

### Characteristics of respondents

A total of 347 respondents, 61% were female and 60.8% were Malays. Respondent’s age were above 18 years old, with the mean age of 42 years old. Majority of the respondent (78.1%) were from lower education level which defined in this study from no formal education until secondary school. Most of the respondent were employed (59.7%), married (62.5%) and have no chronic medical illness (69.7%). The mean household income was MYR 1737, which lower or equal value of mean classified as lower income and higher value classified as higher income (Table 1).

**Table 1:** Socio demographic characteristics of respondents (*N* = 347)

Variables	Mean (SD)	n (%)
Gender		
Female		212 (61.1%)
Male		130 (37.5%)
Missing		5 (1.4%)
Age group (year)		
Mean	42.2 (11.26)	
18–28		37 (10.7%)
29–38		89 (25.6%)
39–48		110 (31.7%)
49–58		62 (17.9%)
59–68		33 (9.5%)
> 69		1 (0.3%)
Missing		15 (4.3%)
Ethnic		
Malay		211 (60.8%)
Chinese		7 (2.0%)
Indian		20 (5.8%)
Others		103 (29.7%)
Missing		6 (1.7%)
Education level		
No formal education		7 (2.0%)
Primary school		51 (14.7%)
Secondary School		213 (61.4%)
College / University		35 (10.1%)
Missing		41 (11.8%)
Marital status		
Single		33 (9.5%)
Married		217 (62.5%)
Divorced/ Separated		88 (25.4%)
Missing		9 (2.6%)
Place of living		
Rural		177 (51.0%)
Urban		148 (42.7%)
Missing		22 (6.3%)
Employment status		
Unemployed		140 (40.3%)
Employed		207 (59.7%)
Total household income (MYR)		
Mean	1736.81 (2382.14)	
< 1000		159 (45.8%)
1000 – 1999		88 (25.4%)
2000 – 2999		48 (13.8%)
3000 – 3999		17 (4.9%)
> 4000		35 (10.1%)
Chronic medical illness		
Present		105 (30.3%)
Absent		242 (69.7%)

### QOL by EQ. 5D utility value

A total of 283 EQ. 5D score sets have been analysed and converted into utility value. 64 respondents had not reported a full score; thus, it was not counted for analysis. A total of 36 of respondents reported full health with having no problem in all the EQ. 5D domains. The utility value mean score was 0.836 (Standard Deviation, SD: 0.12).

Table 2 showed factors that had significant association with low QOL. Male had 1.8 higher risk [OR 1.79 95%CI (1.07, 2.94)], single or no partners [OR 1.72 95%CI (1.05,2.82)], household income of less than RM1737 had 1.7 higher risk [OR 1.72 95%CI (1.03,2.88)] and present chronic illness had 4.5 higher risk for low QOL [OR 4.54 95%CI (2.67,7.71)].

**Table 2:** Bivariate analysis of LSE population factors towards QOL Utility value

Independent variables	QOL utility value		Χ^2^	*p*-value	OR (95% CI)
	Low	High			
Gender					
Female	78 (45.3)	94 (54.7)	5.06	0.025	0.56 (0.34–0.93)
Male	34 (31.8)	73 (68.2)			
Age (years)					
≤ 42	43 (34.4)	82 (65.6)	2.84	0.092	0.66 (0.40–1.07)
> 42	67 (44.4)	84 (55.6)			
Ethnic					
Non-Malay	43 (43.0)	57 (57.0)	0.61	0.433	1.22 (0.74–2.01)
Malay	68 (38.2)	110 (61.8)			
Education					
Low	94 (43.3)	123 (56.7)	3.86	0.051	2.21 (0.98–4.98)
High	9 (25.7)	26 (74.3)			
Employment					
Employed	65 (36.9)	111 (63.1)	1.74	0.187	0.72 (0.44–1.17)
Unemployed	48 (44.9)	59 (55.1)			
Marital status					
Single	50 (48.5)	53 (51.5)	4.64	0.031	1.72 (1.05–2.82)
Married	62 (35.4)	113 (64.6)			
Living place					
Urban	49 (40.5)	72 (59.5)	0.00	0.980	1.00 (0.61–1.64)
Rural	59 (40.4)	87 (59.6)			
Household income					
(MYR)	82 (44.3)	103 (55.7)	4.30	0.038	1.72 (1.028–2.88)
≤ 1737	31 (31.6)	67 (68.4)			
> 1737					
Medical status					
Present	59 (64.1)	33 (35.9)	33.28	0.001	4.54 (2.67–7.71)
Absent	54 (28.3)	137 (71.7)			

As shown in Table 3 only chronic medical illness was significant as QOL determinant in the binary logistic regression model. Present of chronic medical illness has 4.1 higher incidence of low QOL [Adjusted OR 4.15 95%CI (2.42,7.13)] compared to absent illness. The goodness of fit of the model was assessed by Hosmer and Lemeshow test (*p*-value 0.912), and the model correctness was 70.4%.

**Table 3:** Logistic Regression analysis for determinants of LSE population QOL by EQ5D

Factors	Wald	*p*-value	Adjusted Odds Ratio (AOR)	95% CI
Gender				
Female	1.49	0.222	1.43	0.81–2.54
Male			1.00	
Marital status				
Single	0.58	0.443	1.25	0.71–2.23
Married			1.00	
Household income				
Low income	1.91	0.167	1.49	0.85–2.61
High income			1.00	
Chronic medical illness		0.001	4.15	2.42–7.13
Present	26.68			
Absent			1.00	

Significant *p*-value < 0.05

### Health status by VAS

The mean score for health status by EQ-VAS was 7.36 (SD: 2.1). Table 4 showed that factors that associated with poor health status were female [OR 1.82 95%CI (1.08.3.07)], lower education level [OR 3.03 95%CI (1.30,7.05)] and present chronic medical illness with 2.6 higher risk of having poor health [OR 2.63 95%CI (1.51–4.58)].

**Table 4:** Bivariate analysis of LSE population factors towards EQ-VAS

Independent variables	Visual analogue score (EQ-VAS)		Χ^2^	*p*-value	OR (95% CI)
	Poor	Good			
Gender					
Female	76 (50.3)	75 (49.7)	5.14	0.023	1.82 (1.08–3.07)
Male	35 (35.7)	63 (64.3)			
Age (years)					
≤ 42	50 (43.5)	65 (56.5)	0.16	0.9	0.97 (0.58–1.60)
> 42	58 (44.3)	73 (55.7)			
Ethnic					
Non-Malay	42 (48.8)	44 (51.2)	0.96	0.326	1.30 (0.77–2.19)
Malay	69 (42.3)	94 (57.7)			
Education					
Low	96 (49.2)	99 (50.8)	7.10	0.008	3.03 (1.30–7.05)
High	8 (24.2)	25 (75.8)			
Employment					
Employed	67 (42.4)	91 (57.6)	0.87	0.351	0.78 (0.47–1.30)
Unemployed	46 (48.4)	49 (51.6)			
Marital status					
Single	42 (47.7)	46 (52.3)	0.56	0.453	1.22 (0.72–2.06)
Married	68 (42.8)	91 (57.2)			
Living place					
Urban	50 (45.9)	59 (54.1)	0.07	0.784	1.07 (0.064–1.78)
Rural	56 (44.1)	71 (55.9)			
Household income					
(MYR)	68 (43.9)	87 (56.1)	0.10	0.750	0.92 (0.55–1.53)
≤ 1737	45 (45.9)	53 (54.1)			
> 1737					
Medical status			11.98	0.001	2.63 (1.51–4.58)
Present	46 (61.3)	29 (38.7)			
Absent	67 (37.6)	111 (62.4)			

significant *p*-value < 0.05

Multivariable analysis by all significant factors has shown that all three factors were fitted in the model which include gender, education level and chronic medical illness status. Female had 1.9 higher risk to have poor health compared to male [OR 1.94 95%CI (1.09,3.44)]. Low SES population with lower education and present chronic medical illness had 3 times [OR 3.07 95%CI (1.28–7.34)] and 2.5 times higher risk [OR 2.53 95%CI (1.39–4.61)] of having poor health status, respectively as stated in Table 5. The model correctness was 64.3% (cut of point 0.5) and goodness of fit of the model by Hosmer and Lemeshow test (*p*-value 0.283).

**Table 5:** Logistic Regression analysis for determinants of LSE population health status by VAS

Variable QOL utility value	Wald	*p*-value	Adjusted Odds Ratio (AOR)	95% CI
Gender				
Female	5.19	0.023	1.94	1.09–3.44
Male			1.00	
Education				
Lower education	6.35	0.012	3.07	1.28–7.34
Higher education			1.00	
Chronic medical illness				
Present	9.27	0.002	2.53	1.39–4.61
Absent			1.00	

## Discussion

The two outcomes measured aimed to characterize and give the causal factors of good quality of life and health status of the lower household income population in Malaysia. Economic challenges and stability of developing country might affect this group of population in many aspects. Rising cost of living including cost of foods, housing, and childcare were some of the concerns that has been raised up from this population. Targets and plans in the Eleventh Malaysian plans to help in up-lifting the economic and income level of this population into the middle-income group, has been appreciated and benefited to them.

Within the Malaysian LSE population, 30.3% of them having chronic medical illness which mainly the metabolic diseases e.g. diabetes mellitus, hypertension and high cholesterol level. These has been supported with National Health and Morbidity Survey 2015 for Non-Communicable Diseases (NCDs), where 30% of Malaysian population were hypertensive and 3.5 million with Diabetes, while 47.7% were having high cholesterol level. Given that, people with lower socioeconomic level were the most affected group by NCDs in Asian Pacific Region as they have poor access to health facilities, policies and legislation in reducing NCDs [23]. The present of chronic medical illness had significantly affect their health status and quality of life due to disease complications, limitations or acute problems, and this has been proved by other studies [24–26]. A comparison of hypertensive respondents with higher income with good social support has shown better health status and quality of life [27].

Different countries have different value set when measuring HRQOL of the population according to their different sociocultural characteristics, thus giving different comparable factors [28, 29]. A study in China, showed a significant impairment on both component of physical and mental aspect of HRQOL among the respondents with household income below the poverty line which supported findings from this study [30]. Aging process involved physical and mental status of an individual, which may affect the health and well-being [31], subsequently giving impact to the quality of life. But, the age factor of over 40 years old in this study has no effect on quality of life and health status. Similarly, the economy and financial aspect of an individual with chronic disease were independently associated with low HRQOL [32]. Level of education was a predictor for having poor health status or lower mean score in VAS compared to people with tertiary education level. Better formal education had giving better QOL [33, 34]. Sociocultural differences of ethnicity and economic background has influenced the health and quality of life in China [35].

Variance of demographic, social, economic and environments of the populations around the world with different culture and ethnics, in which comparisons of similarity on health and quality of life hardly to be made and discussed. Overview of HRQOL and health status among LSE population in Malaysia were fairly explored, with a better economic growth in near future, different aspects influenced HRQOL should be determined. It was noted that the target participants were from different regions including persons with disabilities and orang asli (aborigine) group may have other potential limitations, which may effects the overall findings in total score of HRQOL. In addition the dimensions of EQ. 5D include mobility, pain discomfort and anxiety whereas these aspects is one of the limitations especially in literacy skills (among these marginalised group) to answer the self-administered questionnaire that affects the total scoring. It is recommended for future research to enhance the recruitment of persons with disabilities and orang asli (aborigine) group either to do in exploratory or qualitative study.

There were some limitations in this study. Cross sectional study design could not give the causal relationship between the factors and HRQOL. The small number of respondents and significant percentage of missing data may give impact to the study analysis, and self-reported questionnaire could give possible reporting bias in this study. For future study, we suggest on having a larger sample size and to conduct an interview-based data collection. We also recommend analysing specific medical illness or other sociocultural factors that were not studied in this research.

## Conclusion

This study has demonstrated an assessment of HRQOL by EQ. 5D and VAS among low SES population in Malaysia that was presented by the lowest household income group. We found that factors such as differences in sociodemographic, socioeconomic and medical illness status were associated with the HRQOL of the respondents. From this study, absent chronic medical illness was factor related with high HRQOL and good health status among LSE population. External and supporting factors such as economic stability and good health condition could help in improving well-being and quality of life of the poorer group. It is recommended for future research to enhance the recruitment of B40 group either to do in exploratory or qualitative study.

## Abbreviations

B40: Below 40% or Bottom 40%
EQ 5D: EuroQol five Dimension
EQ-VAS: EuroQol Visual Analogue Score
HRQOL: Health Related Quality Of Life
LSE: Low Socioeconomic
M20: Middle 20%
QOL: Quality Of Life
RM: Ringgit Malaysia
SES: Socioeconomic Status
T20: Top 20%
VAS: Visual Analogue Score

## References

[1] Population and Demography (2016). Department of Statistic of Malaysia.

[2] Economic Planning Unit (EPU) (2017). Socioeconomic statistic.

[3] Berkman LF, Kawachi I, Maria M (2014). Socioeconomic status and health. Social Epidemiology.

[4] Australian government in measuring socioeconomic status discussion paper. 2012. Available from: https://cgc.gov.au/attachments/article/173/2012-03%20Measuring%20Socio-Economic%20Status.pdf. Accessed 20 Apr 2017.

[5] Economic Planning Unit (EPU). Eleventh Malaysian Plan: improving well-being for all. Available from: http://www.epu.gov.my/en/rmk/eleventh-malaysia-plan-2016-2020. Accessed 10 Apr 2017.

[6] World Health Organization (WHO). World Health Organization Quality of Life (WHOQOL). Available from: http://www.who.int/mental_health/publications/whoqol/en/. Accessed 20 Apr 2017.

[7] Centre for Disease Control (CDC). Health-Related Quality of Life (HRQOL). Available from: https://www.cdc.gov/hrqol/index.htm. Accessed 20 Apr 2017.

[8] What is EQ 5D. Available from: http://www.euroqol.org/. Accessed 20 Apr 2017.

[9] Shafie AA, Hassali MA, Liau SY (2011). A cross-sectional validation study of Eq-5d among the Malaysian adult population. Qual Life Res.

[10] Braveman P (2011). Accumulating knowledge on the social determinants of health and infectious disease. Public Health Rep.

[11] Sommer I, Griebler U, Mahlknecht P, Thaler K, Bouskill K, Gartlehner G (2015). Socioeconomic inequalities in non-communicable diseases and their risk factors: an overview of systematic reviews. BMC Public Health.

[12] Zainal NR, Kaur G, Ahmad NA, Khalili JM (2012). Housing conditions and quality of life of the urban poor in Malaysia. Procedia Soc Behav Sci.

[13] Saleem F, Hassali MA, Shafie AA, Haq N, Farooqui M, Aljadhay H (2015). Pharmacist intervention in improving hypertension-related knowledge, treatment medication adherence and health-related quality of life: a non-clinical randomized controlled trial. Health Expect.

[14] Mahadeva S, Yadav H, Everett SM, Goh K-L (2012). Economic impact of dyspepsia in rural and urban Malaysia: a population-based study. J Neurogastroenterol Motil.

[15] Louise L, Donna R (2013). Mapping to obtain EQ-5D utility values for use in NICE health technology assessments. Value Health.

[16] Portal Pembasmian Kemiskinan eKASIH. Available from https://ekasih.icu.gov.my/Pages/InfoeKasih.aspx. Accessed 22 Apr 2017.

[17] Varatharajan Shanthi, Chen Won-Sun (2011). Reliability and Validity of EQ-5D in Malaysian Population. Applied Research in Quality of Life.

[18] The Euro group (1990). EuroQol - a new facility for the measurement of health-related quality of life. Health Policy.

[19] Yusof FAM, Goh A, Azmi S (2012). Estimating an Eq-5d value set for Malaysia using time trade-off and visual analogue scale methods. Value Health.

[20] Versteegh MM, Rowen D, Brazier JE, Stolk EA (2010). Mapping onto Eq-5 D for patients in poor health. Health Qual Life Outcomes.

[21] Sobocki P, Ekman M, Agren H, Krakau I, Runeson B, Martensson B (2007). Health-related quality of life measured with EQ-5D in patients treated for depression in primary care. Value Health.

[22] Luo N, Chew LN, Fong KY, Koh DR, Ng SW, Yoon KH (2003). A comparison of the EuroQol-5D and the health utilities index mark 3 in patients with rheumatic disease. J Rheumatol.

[23] Tuckett A, Henwood T, Oliffe JL, Kolbe-Alexander TL, Kim JR (2016). A comparative study of Australian and New Zealand male and female nurses’ health: a sex comparison and gender analysis. Am J Mens Health.

[24] Cherepanov D, Palta M, Fryback DG, Robert SA (2010). Gender differences in health-related auality-of-life are partly explained by sociodemographic and socioeconomic variation between adult men and women in the US: evidence from four US nationally representative data sets. Qual Life Res.

[25] Petersson C, Huus K, Enskär K, Hanberger L, Samulesson U, Åkesson K (2016). Impact of type 1 diabetes on realth-related quality of life among 8–18-year-ld children. Compr Child Adolesc Nurs.

[26] Mielck A, Vogelmann M, Leidl R (2014). Health-related quality of life and socioeconomic status: inequalities among adults with a chronic disease. Health Qual Life Outcomes.

[27] Tan Z, Liang Y, Liu S, Cao W, Tu H, Guo L, Xu Y (2013). Health-related quality of life as measured with Eq-5d among populations with and without specific chronic conditions: a population-based survey in Shaanxi Province, China. PLoS One.

[28] Xu X, Rao Y, Shi Z, Liu L, Chen C, Zhao Y (2016). Hypertension impact on health-related quality of life: a cross-sectional survey among middle-aged adults in Chongqing, China. Int J Hypertens.

[29] Tongsiri S, Cairns J (2011). Estimating population-based values for Eq-5d health states in Thailand. Value Health.

[30] Dolan P, Gudex C, Kind P, Williams A (1995). A social tariff for EuroQol: results from a UK general population survey: University of York, Centre for Health Economics York.

[31] Lam LK, Guo Y, Wong KH, Yu YK, Fung SC (2017). Poverty and health-related quality of life of people living in Hong Kong: comparison of individuals from low-income families and the general population. J Public Health.

[32] Khan RJ, Gebreab SY, Crespo PR, Xu R, Gaye A, Davis SK. Race-specific associations between health-related quality of life and cellular aging among adults in the United States: evidence from the National Health and nutrition examination survey. Qual Life Res. 2017. 1–1. https://www.ncbi.nlm.nih.gov/pubmed/28597109. 10.1007/s11136-017-1610-9 PMC5597687 28597109

[33] Al Hayek AA, Robert AA, Al Saeed A, Alzaid AA, Al Sabaan FS (2014). Factors associated with health-related quality of life among Saudi patients with type 2 diabetes mellitus: a cross-sectional survey. Diabetes Metab J.

[34] Leow MKS, Griva K, Choo R, Wee HL, Thumboo J, Tai ES (2013). Determinants of health-related quality of life (HRQoL) in the multiethnic Singapore population–a national cohort study. PLoS One.

[35] Sun S, Chen J, Johannesson M, Kind P, Xu L, Zhang Y, Burström K (2011). Regional differences in health status in China: population health-related quality of life results from the National Health Services Survey 2008. Health Place.

